# Synthesis of graphene–transition metal oxide hybrid nanoparticles and their application in various fields

**DOI:** 10.3762/bjnano.8.74

**Published:** 2017-03-24

**Authors:** Arpita Jana, Elke Scheer, Sebastian Polarz

**Affiliations:** 1 Department of Chemistry, University of Konstanz, 78457 Konstanz, Germany; 2 Department of Physics, University of Konstanz, 78457 Konstanz, Germany

**Keywords:** graphene, hybrid, nanoparticle, reduced graphene oxide, transition metal oxide

## Abstract

Single layer graphite, known as graphene, is an important material because of its unique two-dimensional structure, high conductivity, excellent electron mobility and high surface area. To explore the more prospective properties of graphene, graphene hybrids have been synthesised, where graphene has been integrated with other important nanoparticles (NPs). These graphene–NP hybrid structures are particularly interesting because after hybridisation they not only display the individual properties of graphene and the NPs, but also they exhibit further synergistic properties. Reduced graphene oxide (rGO), a graphene-like material, can be easily prepared by reduction of graphene oxide (GO) and therefore offers the possibility to fabricate a large variety of graphene–transition metal oxide (TMO) NP hybrids. These hybrid materials are promising alternatives to reduce the drawbacks of using only TMO NPs in various applications, such as anode materials in lithium ion batteries (LIBs), sensors, photocatalysts, removal of organic pollutants, etc. Recent studies have shown that a single graphene sheet (GS) has extraordinary electronic transport properties. One possible route to connecting those properties for application in electronics would be to prepare graphene-wrapped TMO NPs. In this critical review, we discuss the development of graphene–TMO hybrids with the detailed account of their synthesis. In addition, attention is given to the wide range of applications. This review covers the details of graphene–TMO hybrid materials and ends with a summary where an outlook on future perspectives to improve the properties of the hybrid materials in view of applications are outlined.

## Review

### Introduction

Graphene consists of a single layer of carbon in a two-dimensional (2D) lattice. It is a densely packed, atomically thin layer of sp^2^ hybridised carbon atoms arranged in a honeycomb network. Since the first report in 2004 [1], graphene has attracted great interest in the scientific community due to its unique properties such as superior charge carrier mobility, high transparency, excellent flexibility, and extraordinary electronic quality, and its superior thermal and mechanical properties [2–3]. Graphene exhibits high mechanical strength (>1060 GPa) and an exceptional Young’s modulus of 1 TPa [4]. Furthermore, single layer graphene is the strongest material ever tested [5]. It also exhibits excellent thermal (≈5000 W·m^−1^·K^−1^) [6] and electrical conductivity (up to 6000 S·cm^−1^) [7] and high theoretical specific surface area (2630 m^2^·g^−1^) [8]. Graphene is highly optically transparent (transmittance ≈97.7%) with absorption of <2.3% for visible light [9] and negligible reflectance (<0.1%), practically independent of the wavelength [10]. Due to these superior properties, it holds great promise for potential applications in many technological fields such as nanoelectronics [11], hydrogen storage, supercapacitors [12] and sensors [13]. Despite its many interesting properties, graphene has a strong tendency to agglomerate due to van der Waals interaction between the graphene layers, which inhibits its application is various fields – this drawback can be eliminated by hybridising graphene with NPs. Graphene is a zero band gap material and the main disadvantage of using graphene alone as a catalyst is its susceptibility to oxidative environments.

In the last few decades, the synthesis of transition metal oxide (TMO) NPs has attracted much attention, providing the advantages of controlled shape, size, crystallinity and functionality, as well as being ecologically benign, corrosion resistance, easily scalable and relatively cost effective [14–16]. In particular, among all the TMO NPs, titanium dioxide [17], manganese oxide [18], iron oxide [19] and zinc oxide [20] have attracted the most attention due to their particular interesting and advantageous properties. By changing the reaction conditions in the synthesis process, such as reaction time, temperature, and precursor concentration, the morphology and size of these TMO NPs can be tuned, resulting in different and unique properties. These materials have a wide range of applications in electronics, optics, electrochemical, solar energy harvesting and so on. In order to further enhance their properties, TMO NPs have been hybridised with graphene where some disadvantages of the NPs can be offset by graphene. The common drawbacks of semiconductor NPs include their relatively low conductivity and high recombination rate of photogenerated electron–hole pairs. Additionally, the NPs result in a large volume expansion during the Li insertion–extraction process in LIB applications which hamper their use in applications such as energy storage, sensing, advanced catalysis, solar cells, diodes and also in biometrics. Thus, strongly coupled graphene–NP hybrid systems appear promising to overcome these problems.

The interest in preparing graphene–TMO NPs hybrids is increasing enormously due to the peculiarities in combining the required properties of building blocks for a specified application. In the recent review by Khan et al., the synthesis, properties and applications of graphene–metal oxide composite NPs are discussed in detail [21]. The review by Yin et al. focusses on graphene–NP-based hybrid sensors [22], while Xiang et al. review the state of the art in graphene–semiconductor photocatalysts [23]. In this review, we comprehensively discuss the different methods for the synthesis of graphene and graphene–NP hybrid systems, but do not cover graphene–NPs composite materials. We then separately review the synthesis, the morphology of graphene–TMO NP hybrids of first row, d-block element oxides, and their applications in various fields.

### Material property requirements for specific applications

Graphite is commercially used as an anode material for LIBs due to its large lithium storage capacity of 372 mAh·g^−1^. However, this is not sufficient for applications requiring high energy capacity. Single layer graphene has a high theoretical lithium storage capacity of 744 mAh·g^−1^, but graphene has a tendency to stack due to van der Waals interactions between graphene layers [24]. The incorporation of TMO NPs inhibits the aggregation of graphene layers [25]. Poizot et al. first introduced the concept of utilising electrodes made of NPs of transition metal (e.g., Co, Ni, Cu, Fe) oxides for LIB applications by using 2 µm cobalt oxide (CoO) particles, achieving an electrochemical capacity of 700 mAh·g^−1^ with 100% capacity retention for up to 100 cycles [26]. For metal oxide in LIB applications, volume expansion occurs during the Li insertion and extraction process, which results in decreased cyclability and rate capabilities [27]. These drawbacks can be overcome by the incorporation of graphene with TMO. As mentioned above, graphene has a relatively large capacity, much higher than commercial graphite. The high conductivity and large surface area of graphene help to maintain the mechanical strength of the hybrid during the Li insertion and extraction process.

Graphene is used as a supercapacitor because of its unique properties, such as high surface area, excellent flexibility, chemical inertness and good electrical conductivity [28]. The practical use of the entire surface area of a graphene sample is difficult so it is often used in combination with an active metal oxide for application as an electrode material in supercapacitors. The main drawback of graphene for optical applications is its zero band gap. However, the development of a heterostructure with a direct band gap semiconductor allows this material to be applied in light emitting diodes (LEDs). In most cases, the other counterpart of graphene–NP hybrids are either transition metal or metal oxide NPs. However, in some cases, depending on the scientific requirements, multicomponent NPs have been also integrated with graphene, but such examples are still very few to date [29].

In a photovoltaic cell, sunlight energy is directly converted to electricity. Graphene and graphene–NP hybrids have been investigated extensively in the field of solar cells because of their unique properties, such as high optical transparency, electrical conductivity, and mechanical flexibility. As mentioned before, graphene is an excellent electron-accepting and electron-transporting material. When graphene is integrated with semiconductor materials, it promotes photogenerated electrons through π–π bond interactions and suppresses the charge recombination in the semiconductor materials [30]. As a consequence, improved photocatalytic properties of the hybrid system have been demonstrated.

### Synthesis of graphene

**Figure 1:**
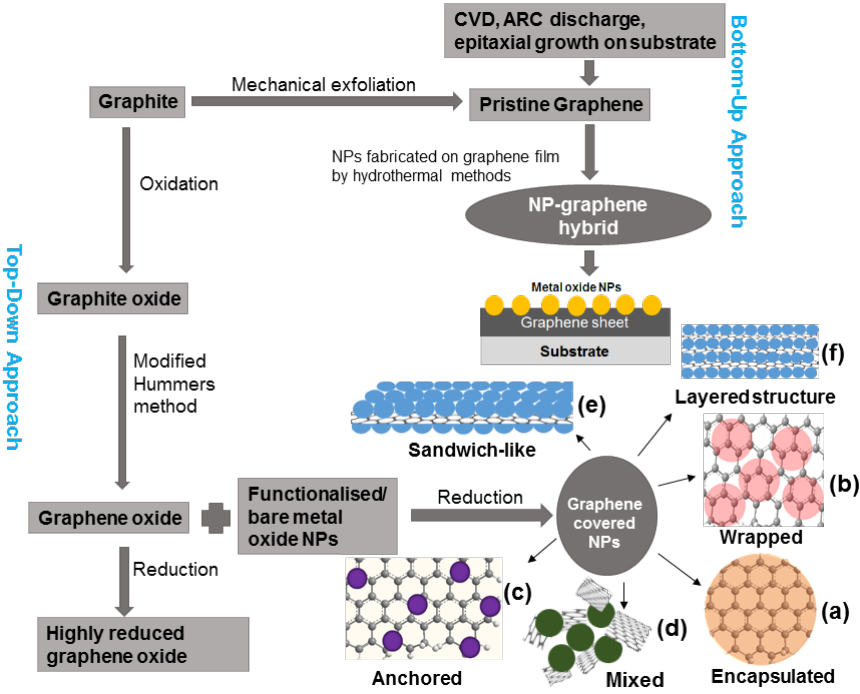
Schematic presentation of methods used for the formation of graphene–NP hybrids and different structures of (a) graphene-encapsulated NPs, (b) graphene-wrapped NPs, (c) NPs anchored to GSs, (d) mixed graphene–NP structures, (e) graphene–NP sandwich structures, and (f) graphene–NP layered hybrids.

The first free-standing single-layer graphene was obtained in 2004 by the isolation of graphene from graphite by micromechanical cleavage [1]. Later, graphene was prepared in bulk from graphite utilising various approaches, including micromechanical exfoliation of pyrolytic graphite [31–33] (the scotch tape method), epitaxial growth [34], chemical vapour deposition (CVD) [35–36], and different chemical functionalisation processes [37–38]. Graphene prepared by the first three processes has the highest quality in terms of structure and properties. The different processes for the synthesis of graphene can be classified into two main categories: bottom-up approaches and top-down approaches (Figure 1). Bottom-up growth of graphene includes micromechanical exfoliation of bulk graphite. The processes included in the bottom-up synthesis of graphene are CVD [39–40], arc discharge [41], and epitaxial growth [42]. Using CVD, graphene and few-layer graphene have been grown on catalytic metal surfaces from carbon containing gasses. In terms of production, the CVD method is used for the production of graphene with a large area with low defect concentration, but in small quantities. Reina et al. have prepared 1- to 12-layer graphene having continuous films with up to ≈20 µm in lateral size by using ambient pressure CVD on polycrystalline Ni; this was transferred to a variety of substrates like SiO_2_/Si or glass [36]. For the preparation of graphene–NP hybrids, bulk quantities of GSs are required. Therefore, for the synthesis of graphene–NP hybrid systems, top-down approaches have been used in most cases.

The most common top-down approach for the synthesis of graphene is the reduction of GO and of graphite fluoride by thermal or chemical methods. The reduction of GO is a low cost, facile technique and yields bulk quantities of GSs. GO was first reported 150 years ago [43]. It is now being reinvestigated and receiving intense research interest due to its extensive use as a precursor for the large-scale synthesis of graphene and graphene-based materials. Initially, GO was prepared from inexpensive graphite as a raw starting material by the cost effective and scalable, modified Hummers method [44]. Using oxidation and exfoliation of this graphite oxide, followed by a reduction process, graphene can be achieved as highly rGO. Like graphite, GO has a layered structure, but the plane of the carbon atoms are heavily decorated by oxygen-containing groups which expand the interlayer distance and make graphite oxide hydrophilic. Exfoliated GO has only one or few layers of carbon atoms like graphene. GO can be reduced to graphene by removing the oxygen-containing groups with the recovery of the conjugated structure. Different reduction processes have been introduced to convert GO to graphene and different reduction processes result in different properties of graphene, which in turn affect the final product and also the performance of the material [45]. Reduced GO is chemically derived graphene, which is also referred to as functionalised graphene [46], chemically modified graphene [47], rGO [48], chemically converted graphene [49], or reduced graphene [50]. For the chemical reduction of GO, hydrazine monohydrate and dimethylhydrazine have been used extensively as they do not react with water and have the attractive option for reducing GO in an aqueous dispersion [51–53]. Though hydrazine effectively removes the oxygen functional group from GO, it also introduces heteroatom impurities such as N_2_ which form amines, hydrazones or other similar functional groups covalently attached on the sheet of graphene [54]. NaBH_4_ has been demonstrated as being more effective than hydrazine as a reducing agent of GO. NaBH_4_ is more effective at reducing the C=O group, while it has moderate efficiency in the reduction of epoxide and carboxylic acid [55]. Other reductants used for the chemical transformation of GO to graphene are hydroquinone [56], gaseous H_2_ [57], alkaline solution [58–59], and ascorbic acid [60]. Despite the chemical reagent reduction, other reduction processes are used for the conversion of GO to graphene, e.g., microwave irradiation [61–62], electrochemical reduction [63–64], thermal annealing [46,65], photocatalytic reduction [66], solvothermal reduction [67–68], thermal deoxygenation [69], or chemical deoxygenation [70]. In terms of electrical conductivity, the quality of reduced graphene is lower than the GS prepared by CVD. The intrinsic quality of CVD graphene films makes them an excellent candidate for optoelectronic and electronic applications. In brief, the reduction method fits better for the production of small GSs, and for larger GSs, the CVD process is more efficient. Thus, depending on the application need of graphene, a suitable fabrication method could be selected.

### Synthesis and architecture of graphene–NP hybrids

For the preparation of graphene–NP hybrids, bulk quantities of GSs are required. So, for the synthesis of graphene–NP hybrid systems, in most cases, top-down approaches have been used. Different architectures of graphene–NP hybrids can be prepared by this one-pot synthesis method [71]. In another process for the synthesis of graphene–NP hybrids, graphene or NPs could be chemically functionalised firstly and then NPs could be conjugated to the graphene surface by covalent or noncovalent interaction [72–73]. Graphene is flexible and has a unique 2D sheet-like structure. These sheets can be easily used to wrap or encapsulate NPs with diameters from the nanometre to even the micrometre range. The encapsulated structure is comprised of a single NP encapsulated by a single GS (Figure 1a), whereas a wrapped structure refers to the case where more than one NP is covered by multiple GSs (Figure 1b) [74]. Graphene-encapsulated and -wrapped structures have advantages compared to bare NPs, including reduced NP agglomeration, as well as increased electrical, electrochemical and optical properties. Encapsulating NPs with graphene increases the surface-to-volume ratio that is available for sensing [75]. Furthermore, the presence of graphene results in extremely high carrier mobility, high carrier density, and low intrinsic noise for better detection by virtue of the high signal-to-noise ratio. In the anchored structure, electroactive NPs are anchored on the GS (Figure 1c) [76], and in the mixed structure, the graphene and NPs are synthesised separately and mixed mechanically for application needs in various fields (Figure 1d) (although mainly used for electrode preparation in LIB applications). In the sandwich structure, graphene is used as a template to generate the active NP/graphene sandwich structure (Figure 1e) [77], and in the layer structure, graphene and NPs (Figure 1f) [27] are alternated. Most research effort has been directed towards the improvement of the quality of rGO in order to ameliorate the properties of graphene [45]. Here, different semiconductor NPs are integrated for different application aspects. To make the discussion more precise, in the following, we categorise the graphene–TMO semiconductor NP hybrids on the basis of their counterpart material oxide (from titanium to zinc) following the periodic table.

#### Titanium dioxide (TiO_2_)–graphene hybrids

Nanocrystalline TiO_2_ is an interesting material because of its unique optical and electrical properties. It has been used as a heterogeneous photocatalyst and it has outstanding advantages because of its low cost, scalability, nontoxicity, strong photo-oxidising power and stability in oxidative and acidic environments [78]. Extensive research on TiO_2_ nanomaterials has shown that the energy conversion efficiency of the photovoltaic devices that use TiO_2_ NPs critically depends on the morphology and size of the NPs [79–80]. Additionally, in TiO_2_–graphene hybrid systems, the morphology of TiO_2_ plays an important role in various applications.

TiO_2_ anode materials have outstanding cycling stability and almost no volume expansion occurs when TiO_2_ is fully lithiated. As the electrical conductivity of TiO_2_ is low, it has a weak rate capability in electrical devices. The modification of TiO_2_ with conductive materials like graphene improves its electrical performance significantly. The introduction of graphene into TiO_2_ results in increased conductivity of the hybrid material, higher transparency and efficient charge separation of the system which causes enhanced photocatalytic activity and other novel properties [81]. Also, due to its high conductivity, graphene is highly effective in improving the charge/discharge performance of TiO_2_ anodes and is responsible for the improved photocurrent response of the hybrid materials [82].

In most cases, for the formation of TiO_2_–graphene hybrids, TiO_2_ is prepared by hydrolysis of Ti-containing precursors [83–85] as the hydrolysis rate of the Ti(IV) precursor is very rapid, even hydrolysed instantly when exposed to moisture, but it is not easy to control the morphology and structure of the as-prepared TiO_2_ NPs. The fabrication of TiO_2_–graphene hybrid systems results in a strong coupling between the components therefore resulting in superior activity as compared to the individual materials. The resultant hybrid material displays a synergistic effect of accumulated graphene and TiO_2_ NPs, exhibited by the extraordinary physical and chemical properties in comparison with pristine graphene and bare TiO_2_ NPs [86–87].

Different synthesis routes have been introduced; among them, hydrothermal methods are used extensively for the preparation of TiO_2_–graphene hybrid systems. Liang et al. prepared TiO_2_ NCs on the GO sheet by hydrolysis methods and then converted GO to graphene by hydrothermal treatment [88]. A TiO_2_–graphene nanocomposite hydrogel (TGH) was prepared by Zhang et al. by a facile one-pot hydrothermal approach where the spherical nanostructured TiO_2_ NPs were densely decorated onto the GS [89].

**Figure 2:**
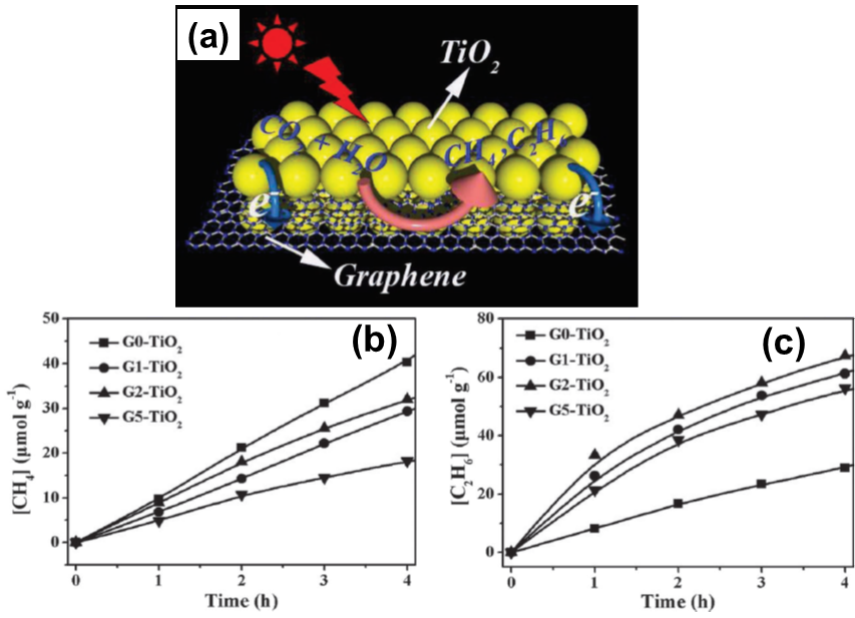
(a) Schematic illustration of the charge separation and transfer in the 2D sandwich-like graphene–TiO_2_ nanostructure system and photoreduction of CO_2_ into CH_4_ and C_2_H_6_. (b) Photocatalytic CH_4_ and (c) C_2_H_6_ evolution amounts for samples G*_x_*TiO_2_ (*x* = 0, 1, 2, 5). The weight contents of graphene designated as *x* (wt %). Reproduced with permission from [90], copyright 2013 Wiley-VCH Verlag GmbH & Co. KGaA.

A 2D graphene–TiO_2_ sandwich structure was prepared by using ethylenediamine/H_2_O solvent in a reduction–hydrolysis technique. The photocatalytic activity of the hybrid has been confirmed by the conversion of CO_2_ to valuable hydrocarbons (CH_4_ and C_2_H_6_) in water vapour (Figure 2). This opens the path for new significant applications of graphene for selectively catalytic C–C coupling reaction [90]. Liu et al. have prepared TiO_2_–graphene hybrid systems using a hydrothermal method. These hybrids have excellent electrochemical performance due to the synergetic effect of well-dispersed TiO_2_ NPs and the conductive graphene network [91]. Nanometre-sized TiO_2_ sheets were prepared on the GSs by using a facial solvothermal synthetic route having improved photocatalytic properties due to the effective charge anti-recombination of graphene and the high catalytic activity of the facets [88].

An anionic surfactant-mediated growth of the self-assembled TiO_2_–graphene hybrid nanostructure synthesis was introduced by Wang et al., which shows enhanced Li-ion insertion/extraction kinetics in TiO_2_, especially at high charge/discharge rates [92]. When an inorganic layer has been intercalated between the two graphene layers it prevents the π–π stacking, creating a functional separation between the individual GSs which allows a periodic modulation of the refractive index and interplay of photon and electron transport. Kumar Manga et al. have prepared multilayer thin films by a self-assembled layer-by-layer technique which consists of alternating titania (Ti_0.91_O_2_) and GO nanosheets. The layer materials are spatially phase segregated to maintain unique 2D characteristics due to the functional separation of the layers at the nanoscale [93]. This hybrid system has potential for achieving the functional separation of charge transport and storage.

A highly efficient, stable, sandwich-structured TiO_2_–Pt–graphene hybrid has been prepared by Xia et al. where the graphene was synthesised by the arc discharge method and the Pt–graphene hybrid electrocatalysts were prepared using a polyol process. This structure exhibits enhanced electrochemical performance due to the strong metal–support interaction and proposed synergetic effect [94]. A molecular grafting process was employed in situ to incorporate GSs in TiO_2_ nanoparticle films for application in dye-sensitised solar cells. The conductivity of the film increases with the incorporation of the GSs, which in turn results in an enhancement of the power conversion efficiency [95].

A unique three dimensional (3D) nanostructure was fabricated by Hu et al. with nanometre-sized TiO_2_ intercalated between graphene layers as pillars which provide a 3D open space with distinct advantages when used as LIB anode materials [96]. (3-Aminopropyl)trithoxysilane was used to functionalise TiO_2_ NPs, and then the TiO_2_ NPs were wrapped by graphene. These hybrids have high potential for photocatalytic application [72]. Zhang et al. have reported graphene-encapsulated TiO_2_ nanospheres as efficient photocatalysts for the decomposition of rhodamine B with an efficiency up to 91% in 90 min, which is much higher than the efficiency of TiO_2_ nanospheres (65%) [97]. The graphene-based nanoarchitecture of TiO_2_ nanospindles [98], TiO_2_ nanorods [92] and TiO_2_ mesoporous [99] shows improved photocatalytic performance via structural optimisation of the architecture.

Although most of the applications of graphene–TiO_2_ make use of its photocatalytic activity, there are also some applications of shape-controlled TiO_2_–graphene hybrids used in pollutant abatement [100], high-performance anodes for microbial fuel cells [101], and self-cleaning applications [102].

#### Vanadium oxide (VO, V_2_O_3_, VO_2_, V_2_O_5_)–graphene hybrids

Vanadium has oxidation states ranging from −1 to +5. Binary vanadium oxides have already been proven as a potential material for studying superconductivity at high pressures and low-dimensional quantum-spin transitions [103]. VO_2_ has two crystalline phases, monoclinic and rutile. The monoclinic form of vanadium(IV) oxide can be transferred to the rutile form by a thermally induced, reversible treatment at 68 °C [104]. VO_2_ (M) behaves as a semiconductor whereas VO_2_(R) behaves as a semimetal. Vanadium pentoxide (V_2_O_5_) has very low electronic conductivity due to its low d-band mobility. It also shows a thermochromic transition at 257 °C and this material is used as a catalyst for industrial processes, gas sensors and in LIBs [105]. Various nanostructures of V_2_O_5_ such as nanotubes, nanowires, nanofibers, nanobelts, and nanorods have been prepared by sol–gel processes, hydrothermal processes [106], electrochemical deposition, and reverse micelle transition [107]. Other vanadium oxides have been studied for their interesting phase change characteristics. Unlike the other oxides of vanadium, VO_2_ is quite stable during lithium intercalation–deintercalation cycles and has been regarded as a promising electrode material for both organic and aqueous LIB owing to high capacity, unique structure and suitable electrode potential. However, its cyclic performance is limited by its high charge-transfer resistance. The incorporation of graphene solves its electrical resistance problem with the VO_2_ electrodes and it acts as a high-performance electrochemical capacitor [108], shows enhanced optical response [109], and represents highly durable electrodes for Li and Na ion batteries [110].

**Figure 3:**
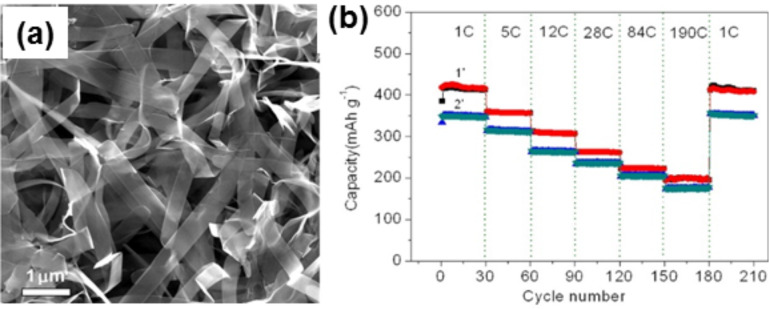
(a) Typical FE-SEM image of a VO_2_–graphene sample prepared by hydrothermal synthesis and reduction of a V_2_O_5_–graphite oxide composite for 1.5 and 12 h at 180 °C, demonstrating the formation of numerous ribbons with a width of 200–600 nm and length of several tens of micrometres. (b) Rate capacities of VO_2_–graphene architectures with different VO_2_ contents, measured for 30 cycles at each selected rate from 1 to 190 cycles. Reprinted (adapted) with permission from [111], copyright 2013 American Chemical Society.

Yang et al. have prepared VO_2_–graphene architectures by using graphene as a substrate for the in situ growth of VO_2_ ribbons (Figure 3a) via hydrothermal synthesis and chemical reduction of V_2_O_5_ by GO simultaneously in a Teflon lined autoclave [111]. When this hybrid is used as a cathode material in LIBs, it provides fast charging and discharging capability with long cycle performance (Figure 3b). VO_2_ (M) nanotube–graphene hybrids cathode material is used in LIB, where the VO_2_ nanotubes are wrapped by and trapped between the GS [112]. Although the capacity of this hybrid is good, the rate capability is not.

To improve the rate capability, Nethravathi et al. use a hydrothermal method to prepare N-doped graphene–VO_2_ nanosheet-built 3D flower hybrids [113], where this typical morphology results in a high surface area as well as better electrical contact with the graphene leading to better electrochemical performance. Liu et al. have developed a sol–gel method for the incorporation of GSs into V_2_O_5_ nanoribbons [114]. These sandwiched GSs not only show enhanced electrial conductivity but also preserve the water molecules between the two layers of V_2_O_5_ which facilitates the Li^+^ diffusion, significantly improving the electrochemical performance. V_2_O_5_ quantum dot/graphene is a promising cathode material for use in long-life rechargeable Li batteries [115–116]. Kim et al. have introduced the use of VO_2_–graphene hybrid materials for the fabrication of thermochromic films for energy saving windows. VO_2_ crystals have been prepared on graphene and the graphene-supported VO_2_ is easily transferred to a plastic substrate which enables formation of a new type of flexible thermochromic film [117]. This hybrid film exhibits efficient operation to reduce the in-house temperature under infrared irradiation.

#### Chromium oxide (Cr_2_O_3_)–graphene hybrids

Cr_2_O_3_, a trivalent chromium(III) oxide, is an important industrial material that has been used in abrading agents and pigments. Unlike other TMOs, Cr_2_O_3_ exhibits poor dispersion. It is insoluble in both acidic and alkaline media. The low electrical conductivity of Cr_2_O_3_ inhibits its application for practical use and electrical research fields. The poor dispersion results in a low surface area and a nonuniform porous structure which restricts its supercapacitive performance. Severe volume change happens for Cr_2_O_3_ during the charge–discharge cycles which results in a rapid capacity fading and the end of the cycle life.

Several strategies have been designed to increase the conductivity of the materials and also to improve the volume change. In that context, graphene has been hybridised with Cr_2_O_3_ to improve the properties of the materials and the other strategy is to develop Cr_2_O_3_ materials on the nanoscale. Graphene–Cr_2_O_3_ hybrids have been explored with respect to the oxygen reduction reaction (ORR) [118] and applications in energy storage [119]. ORR is the key step of renewable energy technologies including fuel cells and water splitting. For excellent electrocatalysts, one of the most important factors is long-term running stability. The long term running stability of the Cr_2_O_3_–rGO hybrid makes it a promising catalyst for fuel cells.

#### Manganese oxide (MnO, Mn_2_O_3_, MnO_2_, Mn_3_O_4,_ Mn_2_O_7_)–graphene hybrids

Pyrolusite (MnO_2_), hausmanite (Mn_3_O_4_) and bixbyite (Mn_2_O_3_) are important minerals of manganese. These oxides have attracted great attention because of their environmental benignity and the high abundance of Mn in nature. Among all the oxides of manganese, Mn_3_O_4_ has been studied widely as an anode material for LIB to achieve higher specific capacities than graphite [120]. Mn_3_O_4_ has a spinal structure and is a potentially interesting electrode material as an electrolytic supercapacitor because of its low cost, environmental compatibility and intrinsically high capacity [18]. But compared to the other TMOs like Co_3_O_4_ and Fe_3_O_4_, Mn_3_O_4_ is a much less conductive material, so it is a great challenge to explore the Mn_3_O_4_ graphene hybrids in LIB applications. As a bulk material, Mn_3_O_4_ has low electrical conductivity that limits its ability in terms of both capacitance and capacitance retention at high current density. The most and widely used strategy is to combine Mn_3_O_4_ to lightweight and electronically conducting graphene. Graphene–Mn_3_O_4_ hybrids have also been employed as active, stable and low cost cathode electrocatalysts used for high capacity anode materials for LIBs [121–124]. The catalytic performance is associated with the morphology, size and coupling of the hybrid materials. Synergistic catalytic-supported interaction between N-doped rGO and Mn_2_O_4_ is used for the vanadium redox flow batteries [125]. During cycling voltammetry, the almost insulating Mn_3_O_4_ is electrochemically oxidised to the more conductive MnO_2_. This explains the interesting phenomenon of increasing capacitance with cycling [126].

**Figure 4:**
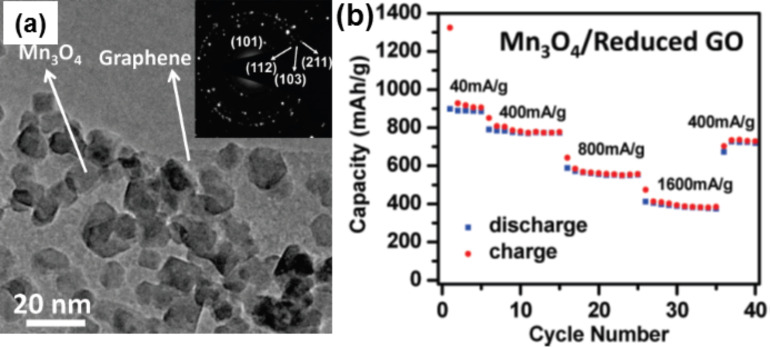
(a) TEM images of the Mn_3_O_4_–rGO hybrid; the inset shows the electron diffraction pattern of the Mn_3_O_4_ NPs on rGO. (b) Capacity retention of the Mn_3_O_4_–graphene-hybrid-based anode at various current densities. Reprinted (adapted) with permission from [136], copyright 2010 American Chemical Society.

The Mn_3_O_4_–graphene hybrid has been also used for the ultrafast oxidative decomposition of methylene blue (MB) [127], for the catalytic decomposition of aqueous organics [128], for carbon dioxide adsorption [129], for ORR [130], for enhancing electrochemical performance for supercapacitors [131–133], and for catalytic oxidation and adsorption of elementary mercury [134]. Bag et al. have prepared Mn_3_O_4_ and N-doped rGO which shows pronounced electrocatalytic activity towards ORR in alkaline solutions [135]. This hybrid can be prepared by different methods such as a two-step solution phase method (Figure 4a) [136], liquid phase deposition [137] or hydrothermal synthesis [138] for growing Mn_3_O_4_ NPs on the GO sheets. The Mn_3_O_4_–graphene hybrid is being explored for high capacity, low cost, nontoxic anode materials for battery applications (Figure 4b). MnO_2_ has a high theoretical capacity of 1232 mAh·g^−1^ deduced from heterogeneous Li_2_O and Mn metal conversion reactions [139]. Yu et al. have prepared free-standing layer-by-layer assembled graphene–MnO_2_ hybrids by an ultrafiltration technique as an anode for LIBs [27]. Dong et al. have prepared 3D hybrids of MnO_2_ graphene foam where the morphology of the hybrids can be readily controlled by the solution acidity [139]. Ultrathin 2D MnO_2_ graphene hybrids form high-performance flexible planar supercapacitors [140]. Graphene-wrapped MnO_2_ is also used for supercapacitor applications [141]. MnO has low conversion potential, low voltage hysteresis (<0.8 V), and high density (5.43 g·cm^−3^). The application of MnO in LIB is a great challenge because the low electric conductivity of MnO results in poor cycling stability and inferior rate capability. A long-term stable, nano-architecture of graphene-supported MnO NPs for LIB applications has been prepared by cycling where the oxidation of Mn(II) to Mn(III) and interfacial lithium storage upon cycling contribute to an enhanced specific capacity [142]. N-doped MnO–graphene prepared by a simple hydrothermal method followed by a heat treatment under ammonia atmosphere, shows a higher capacity and cycle life due to the unique N-doped nanostructure and the efficient mixing with the conducting network [121].

#### Iron oxide (Fe_2_O_3_)–graphene hybrids

**Figure 5:**
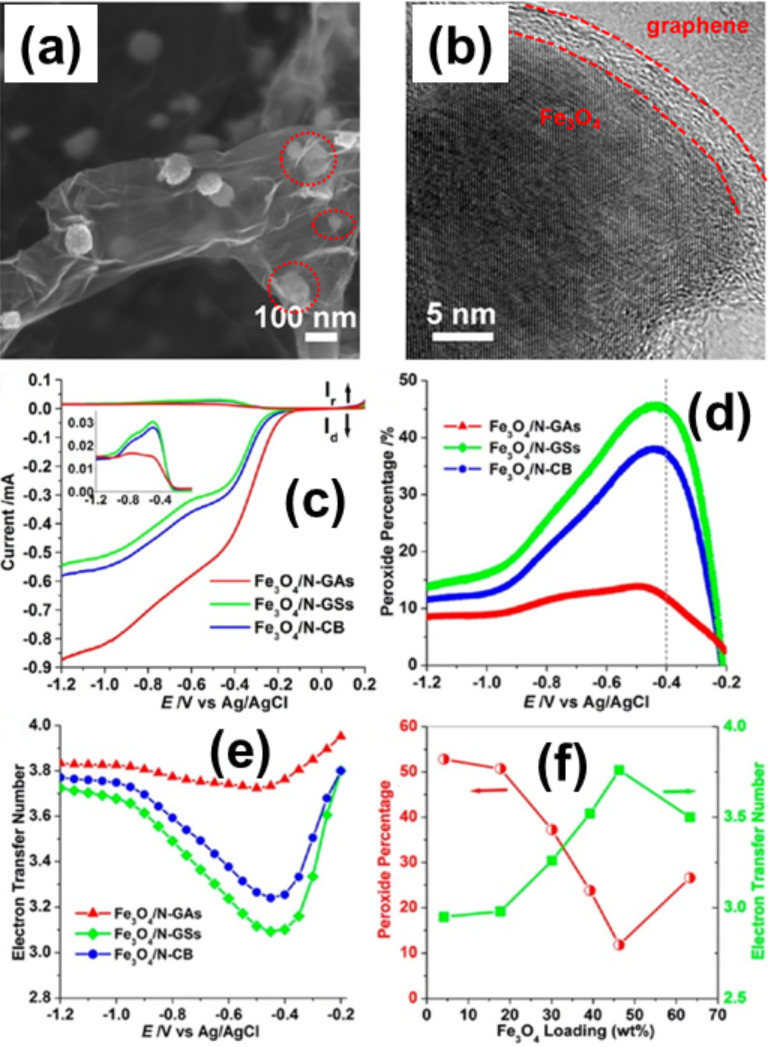
(a) SEM image of N-doped graphene aerogel (N-GA) supported Fe3O4 NPs (Fe3O4/N-GA), where the red markings indicate Fe_3_O_4_ NPs encapsulated in thin graphene layers. (b) HRTEM image of Fe_3_O_4_/N-GA, revealing an Fe_3_O_4_ NP wrapped by graphene layers. (c) The rotating ring disk electrode (RRDE) test of the ORR on Fe_3_O_4_/N-GA, Fe_3_O_4_/N-doped GS (Fe_3_O_4_/N-GSs), Fe_3_O_4_/N-doped carbon black (Fe_3_O_4_/N-CB) in an O_2_-saturated 0.1 M KOH electrolyte at a rotation rate of 1600 rpm. The inset shows the ring current as a function of the electrode potential. (d) Peroxide percentage and (e) electron transfer number of Fe_3_O_4_/N-GAs, Fe_3_O_4_/N-GSs, and Fe_3_O_4_/N-CB as a function of the electrode potential. (f) Peroxide percentage and electron transfer number as a function of Fe_3_O_4_ loading at −0.4 V, as measured with RRDE in an O_2_-saturated 0.1 M KOH electrolyte. Reprinted (adapted) with permission from [147], copyright 2012 American Chemical Society.

Fe_2_O_3_ has the advantages that it can be produced in high abundance, with low cost, and the nontoxicity of Fe results in a reduced environmental concern. Therefore, it is expected to meet the requirements of future energy storage systems. It is an attractive anode material for LIBs as it has a high theoretical capacity (1007 mAh·g^−1^) [143] which is three times larger than that of graphite. During the cycling process, in the host matrix of Fe_2_O_3_ electrode pulverisation and rapid capacity decay happens due to the large specific volume change and particle aggregation. These obstacles can be removed by creating hybrids of Fe_2_O_3_ and graphene which have superior performance regarding flexibility and electrical conductivity [144]. Most of the Fe_2_O_3_–graphene hybrids are prepared by hydrothermal methods without any reducing agent. Tian et al. have prepared α-Fe_2_O_3_ NP anchored graphene hybrid materials by hydrothermal methods which have good cycling performance and enhanced rate capability [145]. A 3D network of free-standing hollow Fe_2_O_3_–graphene has been fabricated by vacuum filtration and a thermal reduction process. This network shows superior electrical performance because the porous nature of the hybrid accommodates the volume change [146]. Wu et al. have use 3D N-doped graphene aerogel (GA) supported Fe_3_O_4_ NPs (Fe_3_O_4_/N-GA) as efficient cathode catalysts for ORR [147]. This Fe_3_O_4_/N-GA material shows excellent electrocatalytic activity for the ORR in alkaline electrodes. It is even larger than N-doped carbon black or N-doped GSs (N-GSs) due to its 3D macroporous structure and high surface area, in addition to exhibiting a higher current density, lower ring current, lower H_2_O_2_ yield, higher electron transfer, and better durability (Figure 5). Chen et al. have prepared graphene/γ-Fe_2_O_3_ hybrid aerogels for the first time which are used for biocatalytic transformation [148]. Fe_2_O_3_ supported on a N-graphene hydrogel was prepared by a facial one-pot hydrothermal method by Ma et al. and is used as an advanced supercapacitor electrode material [149]. Fe_2_O_3_ graphene composites also have significant applications in LIBs [150–153]. Li et al. show the application of monolithic Fe_2_O_3_ graphene hybrids in arsenic removal also due to the self-supported adsorbent properties of the material [154]. α-Fe_2_O_3_–rGO prepared by a hydrothermal method shows good catalytic performance towards the reduction of H_2_O_2_ [155]. Graphene–Fe_3_O_4_ spheres with diameter of about 100 nm were fabricated by a solvent–thermal route and this hybrid shows a homogeneous phase without obvious interface between graphene and Fe_3_O_4_ [156]. Yang et al. have fabricated porous iron oxide ribbons by controlling the nucleation and growth of iron precursors on a graphene surface, which was followed by an annealing treatment and used for high-performance lithium storage [157]. Liang et al. have prepared graphene–Fe_3_O_4_ NP hybrid paper by a filtration process which shows an actuation strain 56% higher than pristine graphene paper [158]. In another work, Liang et al. have prepared free-standing graphene–Fe_3_O_4_ hybrid papers having magnetic-controlled switching performance [159]. One interesting structure was realised using hydrothermal reduction by Wu et al. They prepared Fe@Fe_2_O_3_ core–shell NP–graphene hybrids which show good reversible lithium storage [160]. Another core–shell hollow nanomaterial, a γ-Fe_2_O_3_@graphene hybrid, was prepared through the Kirkendall process by Hu et al. and showed high performance as an anode material for LIBs [161]. The improved performance of the carbon-coated Fe_2_O_3_–graphene hybrids show that the improved performance in LIBs is attributed also to the carbon layer around the Fe_2_O_3_ NPs [146,162]. The thin carbon shells effectively inhibit the direct exposure of encapsulated Fe_3_O_4_ NPs to the electrolyte and preserve the structural and interfacial stabilisation of the NPs. The flexible and conductive graphene and carbon shell around the Fe_3_O_4_ NPs can accommodate the mechanical stress induced by the volume change of the NPs and thus maintain the structural and electrical integrity of the hybrid during the lithiation and delithiation processes [163]. Su et al. reported the change of dynamic behaviour and the conversion mechanism in LIBs by in situ transmission electron microscopy (TEM) characterisation [164].

Fe_2_O_3_ NPs showed volume expansion and morphological changes upon lithiation, and the surface of the electrode was covered by a Li_2_O layer. They also found that the single crystalline Fe_2_O_3_ NPs were converted to polycrystalline NPs and the delithiated product is no longer Fe_2_O_3_ but FeO. A solvothermal process is introduced to construct 3D macroscopic Fe_2_O_3_ nanocube/N-doped graphene aerogels as an anode material for LIB applications [165]. This aerogel structure exhibits excellent rate capability and outstanding long-term cyclic stability at high current densities. Wang et al. have introduced a solvothermal-induced self-assembly approach to construct the monolithic 3D Fe_2_O_3_ and GS hybrids, which have excellent prolonged cycling stability [166]. Geng et al. have prepared Fe_3_O_4_–rGO hybrids by one-pot solution chemistry which have good adsorption capability of various dyes (rhodamine B, rhodamine 6G, acid blue 92, orange (II), malachite green, and new coccine) [167]. These materials could be easily separated from the reaction medium due to the presence of the magnetic Fe_2_O_3_ NPs. In addition, the materials could be regenerated and utilised via a simple annealing treatment.

#### Cobalt oxide (CoO, Co_2_O_3_, Co_3_O_4_)–graphene hybrids

**Figure 6:**
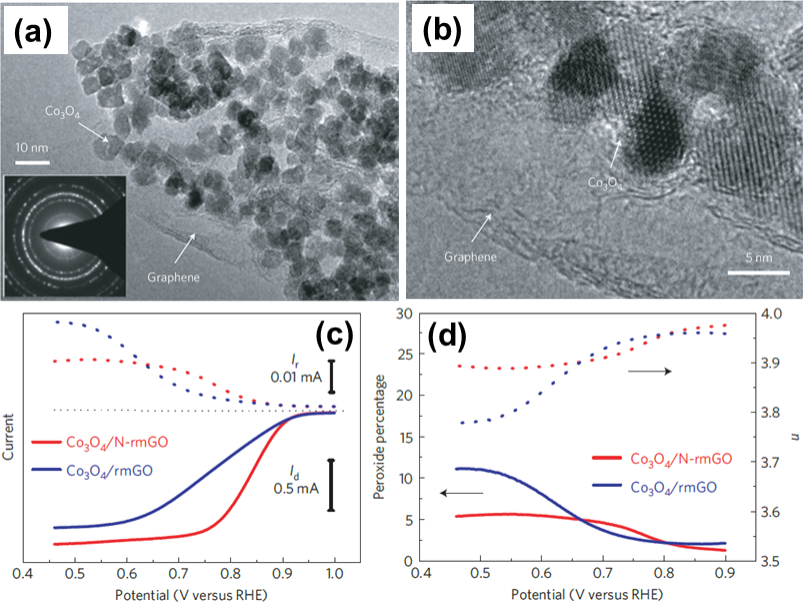
(a) Low magnification and (b) high magnification TEM images of Co_3_O_4_/N-doped reduced mildly oxidised graphene oxide (N-rmGO) hybrid. (c) Assessment of peroxide percentage in ORR catalysed by hybrid catalysts was made from rotating ring-disk electrode voltammograms recorded with Co_3_O_4_/rmGO hybrids (loading ≈0.1 mg·cm^−2^) and Co_3_O_4_/N-rmGO hybrids (loading ≈0.1 mg·cm^−2^) in O_2_-saturated 0.1 M KOH at 1,600 r.p.m. Disk current (*I*_d_) (solid line) is shown on the lower half and ring current (*I*_r_) (dotted line) is shown on the upper half of the graph. The disk potential was scanned at 5 mV·s^−1^ and the ring potential was constant at 1.5 V versus reversible hydrogen electrode (RHE). (d) Percentage of peroxide (solid line) and the electron transfer number (*n*) (dotted line) of Co_3_O_4_/rmGO and Co_3_O_4_/N-rmGO hybrids at various potentials, inferred from the corresponding RRDE data in (c). Reprinted by permission from [170], copyright 2011 Macmillan Publisher Ltd.

Cobaltosic oxide (Co_3_O_4_)–graphene hybrids can be synthesised by solution methods. Like other hybrid TMO–graphene materials, this hybrid is also used as an anode for LIBs [168–169] because it exhibits high oxygen reduction activity. Liang et al. showed that Co_3_O_4_–N-doped graphene exhibits similar catalytic activity like Co_3_O_4_–graphene hybrids but superior stability to Pt in alkaline solution [170] (Figure 6). N-doping of reduced mildly oxidised graphene oxide (rmGO) affords stronger coupling than rmGO and Co_3_O_4_ (Co_3_O_4_/N-rmGO than in Co_3_O_4_/rmGO) due to favourable nucleation and anchor sites for Co_3_O_4_ nanocrystals as N-groups help on rGO. In the ORR, the electronic effect of N-doping of graphene also plays a role. Mao et al. have prepared 3D crumbled cobalt–GO nanostructure hybrids which show both ORR and oxygen evolution reaction (OER) [171]. Wu et al. have prepared 3D Co_3_O_4_/flocculent graphene hybrids on Ni foam for supercapacitor applications as their nanocluster morphology synergistically results in an improved electrochemical performance [172]. Symmetric supercapacitors are based on Co_3_O_4_ NPs on vertically aligned graphene. This hybrid has also been used for nonenzymatic glucose detection in microdroplets [173].

Wang et al. fabricated graphene–Co_3_O_4_ hybrid electrodes constructed on a micropipette tip which can detect nonenzymatic glucose [173]. N-doped graphene/Co_3_O_4_ has been used for selective oxidation of olefins and alcohols [174], LIBs [175], and oxygen reduction [176], and as a water electrolysis catalyst [177]. Co_3_O_4_ NPs were anchored on conducting graphene and used as an anode for high-performance LIBs. This hybrid material exhibited large reversible capacity, excellent cyclic performance and good rate capability [178–179]. Magnetic cobalt NPs anchored on GSs were prepared by a two-step procedure, consisting of code position and thermal treatment [180]. The Co–graphene hybrid showed better catalytic performance as compared to Co only in the degradation of orange II. In another application, atomic cobalt on N-doped graphene has been used for hydrogen generation [181]. Singh et al. have prepared surface-tuned Co_3_O_4_ NPs on N-doped graphene by using a hydrothermal method [182]. Here, the N-doping plays an important role as a supporting material to ensure good dispersion of the oxide NPs and in bringing in favourable activity to the system. They show that this hybrid material reveals an ORR activity that is closely matched with the Pt-supported carbon catalyst in an alkaline medium. The Co_3_O_4_–graphene hybrid possesses catalytic performance for heterogeneous activation of peroxymonosulfate for the decomposition of phenol [183]. Moreover, the performance of the zinc–air battery (ZAB), fabricated by using this hybrid as the cathode, is found to be closely matched with the performance of the systems prepared with Pt/C cathodes. The activity enhancement of the system is due to multiple favourable factors, including the high roughness of Co_3_O_4_, homogeneous dispersion of the NPs, increased surface area of the catalyst, and most importantly, the synergetic interaction between the N-doped graphene and Co_3_O_4_ NPs. In another work, Singh et al. have prepared three different morphologies of Co_3_O_4_ nanocubes, blunt edge nanocubes and spherical particles supported on N-doped graphene by a hydrothermal method [184]. Among those structures, the intermediate architecture, blunt edge nanocubes, show higher catalytic activity towards OER in alkaline medium. This is due to the presence of the low surface energy crystal plane of Co_3_O_4_ NPs and their synergistic interaction between N-doped because graphene helps to tune the properties of the OER electrocatalyst. Yang et al. fabricated graphene encapsulated Co_3_O_4_ NPs which have a high reversible capacity of 1000 mAh·g^−1^ over 130 cycles and is superior to Co_3_O_4_ NPs with respect to capacitor applications [185]. Kumar et al. have prepared graphene-wrapped Co_3_O_4_-intercalated hybrid nanostructures using microwave irradiation [186] and this hybrid shows cycling stability over 10,000 cycles.

CoO attracts extensive interest because of its high lithium storage capacity. CoO–NP-supported GSs effectively circumvent particle cracking, pulverisation, and aggregation upon cycling, thus serving as a high-performance anode material with long cycle life, high reversible specific capacity and excellent rate capability for LIB applications [187]. Self-assembled CoO nanorod clusters were synthesised on 3D graphene through a facile hydrothermal method followed by a heat treatment by Zhu et al. This hybrid exhibited good electromechanical performance [188]. Guo et al. prepared Co/CoO–graphene by self-assembly of Co NPs on the surface of graphene. This hybrid has similar activity as and better stability than commercial Pt NP catalyst supported on carbon (C–Pt) NPs and may serve as an alternative to C–Pt catalysts for the ORR in alkaline solution [189]. Porous graphene wrapped CoO NPs show higher performance in OER due to the porous structure of the hybrid, resulting in high electroactive surface area as well as in strong chemical coupling between the components [190]. An electrochemical nonenzymatic sensor based on a CoO–graphene hybrid is used for enzymeless glucose detection [191] and determination of carbofuran and carbaryl in fruits and vegetables [192]. Instead of using cobalt oxide–graphene hybrids, Yao et al. have synthesised a cobalt hydroxide nanoflake–rGO hybrid by a one-pot hydrothermal method using glucose as a reducing agent for GO reduction [193]. This hybrid shows higher catalytic activity than Co(OH)_2_ for phenol degradation and it takes only 10 min for 100% phenol removal.

#### Nickel oxide (NiO)–graphene hybrids

**Figure 7:**
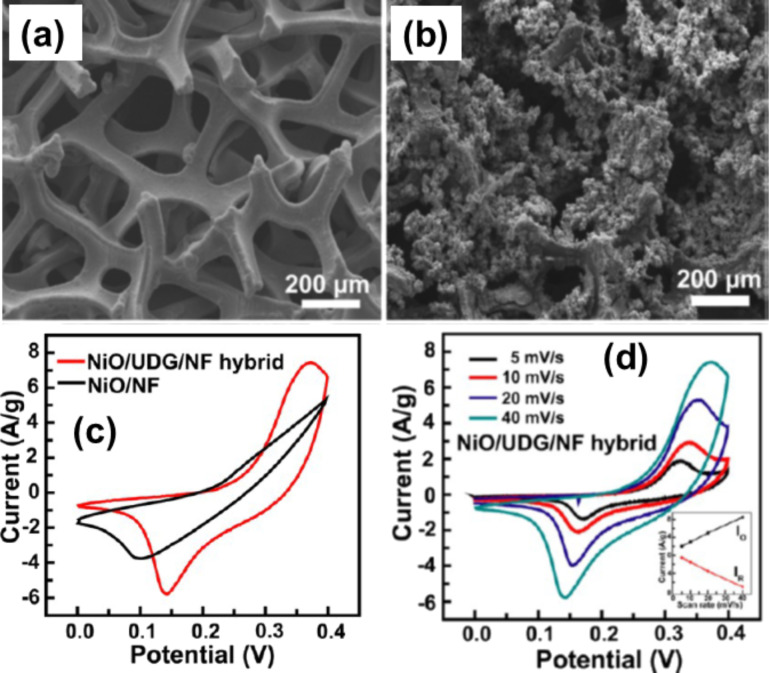
SEM images of (a) 3D ultrathin derived graphene/Ni foam (UDG/NF) scaffold and (b) NiO/UDG/NF hybrid after chemical bath deposition (CBD) growth of NiO nanoflakes. (c) Comparison of cyclic voltammograms of the NiO/UDG/NF hybrid and NiO/NF electrode at a scan rate of 40 mV/s. (d) CV curves of the NiO/UDG/NF hybrid at different scan rates and linear response of the peak current intensity with the scan rate. Reprinted (adapted) with permission from [203], copyright 2014 American Chemical Society.

NiO, a p-type wide band gap semiconductor is extensively used as catalyst, battery cathode, electrochemical capacitor and magnetic material [194]. NiO is considered to be a promising alternative electrode material in redox electrochemical capacitors because of its easy synthesis and high capacitance. Porous NiO–rGO hybrid films were prepared by combination of electrophoretic deposition and chemical bath deposition methods [195–196]. Like other graphene–metaloxide hybrid systems, NiO–graphene hybrids are largely used for LIB applications [197–199]. Monolayer graphene/NiO nanosheet composite materials also have large application for supercapacitors [200–202]. 3D NiO/ultrathin derived graphene hybrids show improved supercapacitor performance compared to bare NiO electrodes (Figure 7) [203]. The introduction of 3D ultrathin derived graphene and Ni foam scaffolds significantly increases the electron transfer rate and also the electrochemical activity of the reversible reaction of Ni^II^ and Ni^III^. The NiO–graphene hybrids show good sensing capability for the reducing gases such as H_2_, NH_3_, H_2_S, NO_2_ [204]. In another work graphene nanosheet–NiO hybrids in combination with DNA are used as the high-performance nonenzymatic glucose sensors [205]. Recently graphene-wrapped NiO hybrids were prepared by Kumar et al. for supercapacitor applications [206]. This hybrid can also be used as an electrochemical pseudocapacitor material for potential energy storage applications [207–208].

#### Copper oxide (Cu_2_O, CuO, CuO_2_, Cu_2_O_3_)–graphene hybrids

Cu nanowire (NW) films and indium tin oxide (ITO) films have comparable sheet resistances and optical transmittance. A few drawbacks of Cu NW films, such as low oxidation resistance or weak adhesion to the substrate, can be compensated for by the addition of graphene. The resulting hybrid films have improved electrical conductivity as they provide 2D pathways for charge transfer [209]. The graphene layer, acting as an oxidation resistance layer, fills the open space by a conductive and transparent film and it protects the NWs from the harsh environment [210].

**Figure 8:**
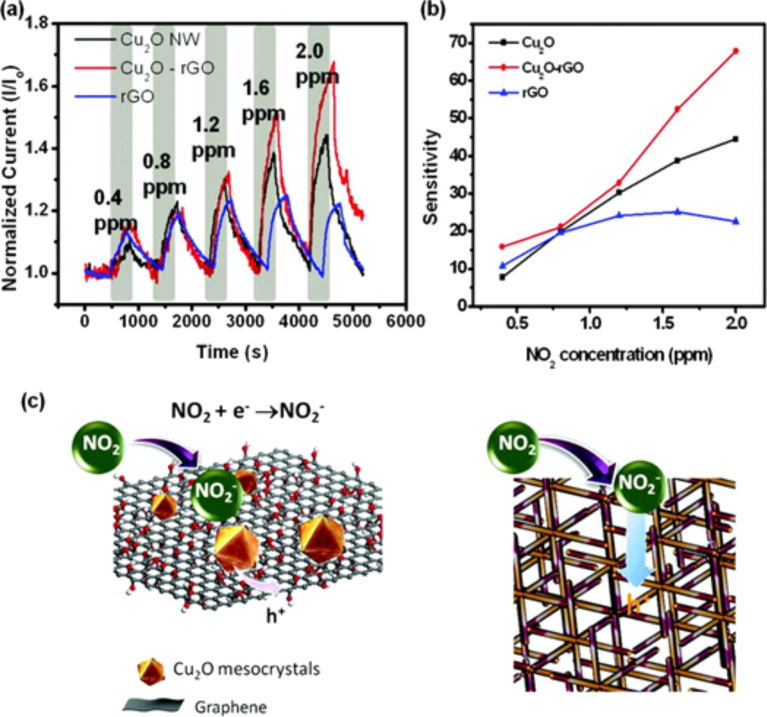
(a) Dynamic response of Cu_2_O NWs, rGO–Cu_2_O, and rGO devices under increasing NO_2_ exposure. (b) The sensitivity of the NO_2_ sensor for the three devices. (c) Schematic for the mechanism of NO_2_ sensing of rGO–Cu_2_O. Reprinted with permission from [214], copyright 2012 American Chemical Society.

Cu NW–graphene has been used as the back contact in thin film CdTe solar cells giving a solar efficiency of 12.1% with excellent thermal stability [209]. Cuprous oxide (Cu_2_O) is a p-type semiconductor and is used for solar energy conversion, as sensors and for photocatalytic degradation. The controlled synthesis of Cu_2_O results in a vast palette of architectures including nanocubes, nanocages, nanowires, solid and hollow spheres. Graphene-wrapped Cu_2_O nanocubes exhibit higher electrocatalytic performance towards electro-oxidation of glucose with lower detection time, and this hybrid has improved electrochemical stability [211]. Ding et al. have prepared Cu_2_O microspheres on rGO by a one-step synthesis which have potential application for nonenzymatic electrochemical glucose and H_2_O_2_ sensors [212]. Ortega-Amaya et al. synthesised GO-coated Cu_2_O and Cu NPs on copper foil by annealing at 200–1000 °C under an Ar atmosphere where the particle phase and particle size and shape strongly depend on the process temperature [213]. Deng et al. synthesised rGO-conjugated Cu_2_O NW mesocrystals by nonclassical crystallisation under hydrothermal conditions [214]. During the synthesis process, Ostwald ripening is responsible for the formation of the NW building block. This porous 3D framework structure, Cu_2_O NW–graphene hybrids, was used as a high-performance NO_2_ gas sensor (Figure 8). Copper oxide (CuO) is also a p-type semiconductor. CuO–graphene composites have also been used as anode material for LIBs [211,215]. Mathesh et al. prepared GO hybrid materials consisting of Cu ions complexed with GO, where Cu^2+^ acts as a bridge, connecting GO sheets and introducing new energy levels along the electron transport pathway thereby opening up possible conduction channels [216]. Singh et al. reported a bipolar, resistive switching device incorporating a copper oxide and multilayer graphene hybrid where the electrical characteristics of CuO–graphene bilayer structure has been modified largely due to the electronic interaction at the hybrid interface. The O_2_ intake capacity of the multilayer graphene results in reversible bipolar resistive switching properties [217]. Zhou et al. prepared graphene-wrapped CuO hybrids by a rapid, facile microwave-assisted hydrothermal method for LIB applications [218]. CuO–graphene nanostructures were used as nonenzymatic glucose sensors [219], humidity sensors [220], for CO_2_ mineralisation [221], as supercapacitors [222], and as pseudo-capacitor electrode materials [223].

#### Zinc oxide (ZnO)–graphene hybrids

**Figure 9:**
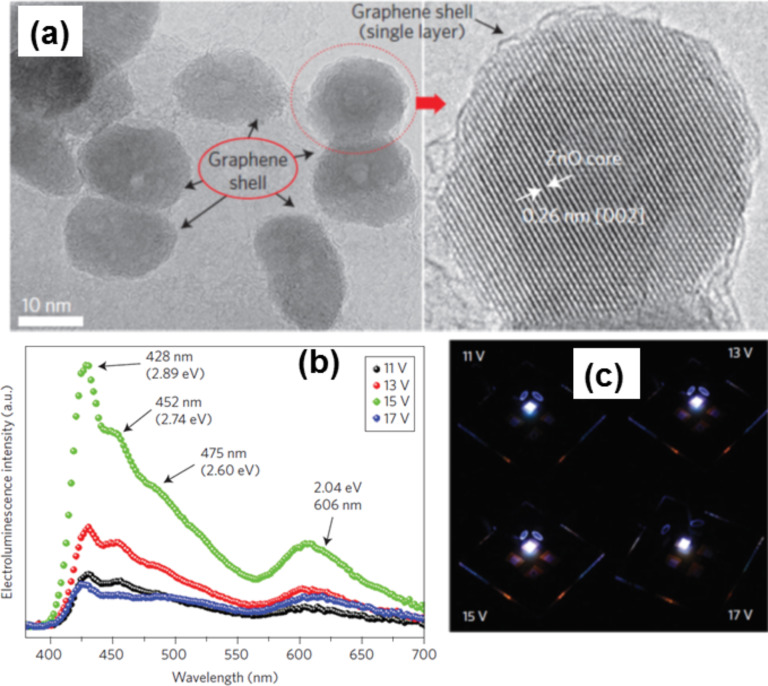
(a) High-resolution TEM images of ZnO quantum dots covered by graphene. The graphene shell layer of the ZnO-core quantum dot covered by graphene looks like a single graphene layer. To the right, a magnified image shows the structure of the ZnO core and graphene monolayer. The ZnO petals grow along the [2] direction. (b) Electroluminescence spectra of the fabricated ZnO–graphene quasi-quantum dot LED device with applied voltage from 11 to 17 V. (c) Photograph of light emission at 11, 13, 15 and 17 V applied voltage bias, respectively. Reprinted by permission from [224], copyright 2012 Macmillan Publisher Ltd.

Most of the reports of graphene hybrid systems are related to ZnO NPs, as it is very easy to control the size and morphology of ZnO NPs as well as the properties of the material. ZnO is an important II–VI semiconductor with large direct band gap of 3.37 eV and large exciton binding energy of 60 meV. It has been extensively studied because of its potential application in solar cells, sensors, diode lasers, piezoelectric devices, as surface acoustic wave propagators, antibacterial agents and ultraviolet light emitters. Graphene-based ZnO hybrids proved to be promising materials having a much wider range of applications in the field of energy conversion and storage, such as catalysts and in optoelectronics. Comprehensive work has been done to prepare graphene-wrapped ZnO NPs for better performance in optoelectronics applications. Single-layer graphene-wrapped ZnO NPs (Figure 9a) have been prepared by a facile technique by Son et al. They show varying electroluminescence spectra with voltage (Figure 9b) which is used in white LEDs (Figure 9c) [224]. In this process, in the solution of di-methyl-formamide (DMF), Zn^2+^ is present along with GO, where the Zn^2+^ reacts with the oxygen functional group of GO, leading to the formation of Zn–O–C bonds. During the reaction, sections of graphene detach from the GO through a layer-by-layer chemical peel-off process (chemical exfoliation) and partially encircle the ZnO NPs. The quasi-core–shell structure of the hybrid was prepared by a one-step chemical method [225].

For the formation of graphene-wrapped ZnO NPs, some other routes have been used, like chemical functionalisation of the ZnO surface. In this process there are three steps: surface modification of NPs mainly by amine groups, coating the NPs with a GO shell, and finally, conversion of GO to graphene by a reduction process. For the amine functionalisation of metal oxide NPs, poly(allylamine hydrochloride) (PAH) [226–227] and (3-aminopropyl)triethoxysilane [228–230] solution has been used widely. In this process, mostly the hydrothermal method has been utilised for the conversion of GO to graphene. Some ZnO–graphene hybrids have been prepared by fabricating vertically aligned ZnO NWs on few or single layers of the graphene substrate. In most of the work, graphene has been prepared by a CVD process with a few layers and large area, whereas the ZnO nanostructures are synthesised on this graphene layer in a tube furnace [231], by hydrothermal methods [227,232–236], MOVPE [237–238], or electrochemical deposition [239]. Li et al. synthesised ZnO–graphene hybrids by a facile freeze-drying treatment and a subsequent heat treatment method by using GO and a zinc hydroxide precursor [240]. In this process, 10 nm ZnO NPs are uniformly anchored on the N-doped graphene matrix to form the hybrid which has enhanced lithium storage capacity.

The optical properties of ZnO NPs change when they form hybrids with graphene. Pure ZnO exhibits a photoluminescence (PL) band at 373 nm, which is due to the exciton recombination corresponding to the band edge emission, and a green emission peak is most commonly observed that arises from the defect of ZnO NPs such as oxygen vacancies, zinc vacancies, oxygen interstitials, and zinc interstitials [241]. Graphene does not show any photoluminescence emission peak as there is no band gap in its electronic state. On the other hand, the intensity of the PL for the band edge of ZnO decreases significantly in the hybrid, presumably due to the enhanced separation rate of photo-induced charge carriers by effective charge transfer from ZnO NPs to the conductive graphene layer [242–246]. The process of electron transfer is temperature dependent. The negative thermal quenching behaviour of ZnO NWs and graphene shells was studied through charging and discharging processes between the two components, and it shows a higher quantum tunnelling probability between graphene and ZnO at an increased temperature [247]. ZnO–graphene quantum dot hybrid structures have potential for use as cathodes for field emission transition applications [248–249]. Kavitha et al. have prepared ZnO–rGO hybrids by a solution precipitation method and a hydrothermal method. The resulting structures show fluorescence quenching properties indicating the energy transfer between the components [250]. They also show that there is a 15-fold enhancement in the nonlinear absorption coefficient of the ZnO–rGO hybrid compared to the bare ZnO. Once the electrons are transferred from ZnO NPs to graphene, they can be recirculated in the graphene channel within their lifetime. If the lifetime is long enough, the electrons form a system which can be utilised as highly sensitive light detectors or sensors [251–252]. Due to this synergetic effect via interfacial charge transfer and inhibition of recombination of excited electrons, this hybrid has wide spread applications in waste water purification by the photocatalytic degradation of dye molecules like MB [253–259], rhodamine B [260], and for the degradation of phenol [261]. This hybrid also has potential for the fabrication of biogas sensors and electrochemical sensors. The ZnO NPs decorated on graphene act not only as spacers between the GSs, but also to enhance the sensitivity towards common industrial toxin gases like CO, NH_3_, NO [262], methane [263], NO_2_ [264], formaldehyde [265–266], H_2_ [267], and also to humidity [268]. Furthermore, this hybrid system is used for the photocatalytic reduction of Cr(VI) [269–270]. The introduction of a noble metal into the ZnO–graphene hybrid produces a synergistic effect, which influences the electronic and chemical distribution of the materials, thereby favouring the adsorption of oxygen species and resulting in high-performance metal-oxide-based sensors. Silver-loaded hierarchical ZnO–rGO hybrids showed preferable detection of acetylene with specific response [271].

ZnO can be used as an inexpensive anode material and has high theoretical capacity (978 mAh·g^−1^) but it has attracted less attention compared to the other metal oxides due to its poor cyclability resulting from huge volume change during charge–discharge cycles. Kushima et al. show that when ZnO NWs are partially lithiated, multiple cracks are induced and divided the NWs into multiple segments. This results in a lithium embrittlement effect in ZnO [272]. Such strain-induced cracking can be minimised by decreasing the particle size to the nanoscale. Sun et al. synthesised ZnO QDs of 2–7 nm on GSs by atomic layer deposition methods. They have enhanced the cycling stability and rate performance in LIBs [273]. ZnO–graphene layered structures also showed improved cycling stability because the flexible graphene layer acts as a buffer to alleviate the volume change and as the separator to reduce the aggregation of the ZnO NPs [274].

Excellent performance in lithium storage capability is observed by incorporation of a conductive nitrogen-doped graphene matrix with ZnO due to a synergistic effect [240]. Boruah et al. used the thermal evaporation technique for the growth of highly dense ZnO nanowires on 3D graphene foam, which has potential application as ultraviolet photodetector [275]. ZnO–graphene hybrids have been largely used for glucose sensing [276], hydrazine sensors [277], in antibacterial applications [278], in electrical and optical devices [279], as multifunctional conductors [280], for photocurrent generation [256], in solar cells [239,281–282], and for UV illumination [283].

#### More than two component TMO–graphene hybrids

Two TMOs can be hybridised with graphene for improved performance which increases the range of potential applications of the hybrid materials. Since the report of Padhi et al., the iron-based nanomaterial, olivine lithium iron phosphate (LiFePO_4_), has attracted increasing attention because it is a promising candidate for cathode materials [284]. LiFePO_4_ is a promising cathode material for LIBs because of its high stability, high power, environmental safety, and low cost. However, the main drawback for LiFePO_4_ battery commercialisation is their poor rate performance (at current >5 C) due to the low electrical conductivity and Li-ion diffusion rate which limits its usage for electrical applications. Those drawbacks are compensated for by hybridising LiFePO_4_ with graphene. Graphene-hybridised LiFePO_4_ has been largely used for LIBs [285–292]. The most interesting properties of LiFePO_4_ are mainly that the operating voltage and theoretical capacity can be increased by hybridisation with graphene. Shi et al. have prepared graphene-wrapped Li_3_VO_4_ microboxes by a one-step in situ hydrothermal method. This hollow structure could relax the stress and strain of Li^+^ insertion/extraction and provides extra space for the storage of Li ion. It also increases the surface area of the material, resulting in improved capacity, rate capability and cycling performance [293].

Much effort has been attributed to the development of Co-based anodes for LIB applications by partially replacing Co^2+^ in Co_3_O_4_ with more ecology friendly and inexpensive materials such as Zn, Cu, Ni, Mg, and Fe. The substitution of environment friendly Mn^2+^ for toxic Co^2+^ in the Co_3_O_4_ spinal lattice results in the formation of Co(II)Co(III)Mn(III)O_4_. Hybridisation of this material with graphene results in further optimisation of catalytic properties. Wang et al. prepared a MnCo_2_O_4_–graphene hybrid by a two-step solution method by using Co(OAc)_2_ and Mn(OAc)_2_ precursors [294]. The hybrid was prepared by hydrolysing the precursors and GO coating followed by a solvothermal process. This hybrid shows improved catalytic properties. MnCo_2_O_4_–graphene has been used as a LiO_2_ battery cathode [295], an oxygen reduction electrocatalyst [29], and in reducing fire hazards of poly(butylene terephthalate) [296]. Mohamed et al. have studied the influence of Co^2+^ replacement in the Co_3_O_4_ matrix by M^2+^ (where M = Mn, Fe, Ni, Zn) for increasing the catalytic performance [297]. For the synthesis of the hybrid, they mainly use hydrothermal methods. Graphene–ZnCo_2_O_4_ hybrids have been used for the ORR [298]. Zhang et al. demonstrated graphene–NiCo_2_O_4_ hybrids as a methanol-tolerant electrocatalyst for the ORR with improved performance [299]. NiCo_2_O_4_–graphene has also been used in electrochemical pseudo-capacitor applications [300]. NiCo_2_O_4_ was anchored on the N-doped graphene prepared by a hydrothermal method, used for LIB applications, where the N-doping further improved the ORR performance in the LiO_2_ batteries [301]. Wu et al. prepared needle-like NiCo_2_O_4_ nanostructures on a 3D graphene foam with in situ deposition by a facile hydrothermal method which is used as an enzyme mimic for glucose and calcium detection [302]. NiCo_2_O_4_ nanoplatelets and graphene hybrids have been used for ORR and OER [303–304].

**Figure 10:**
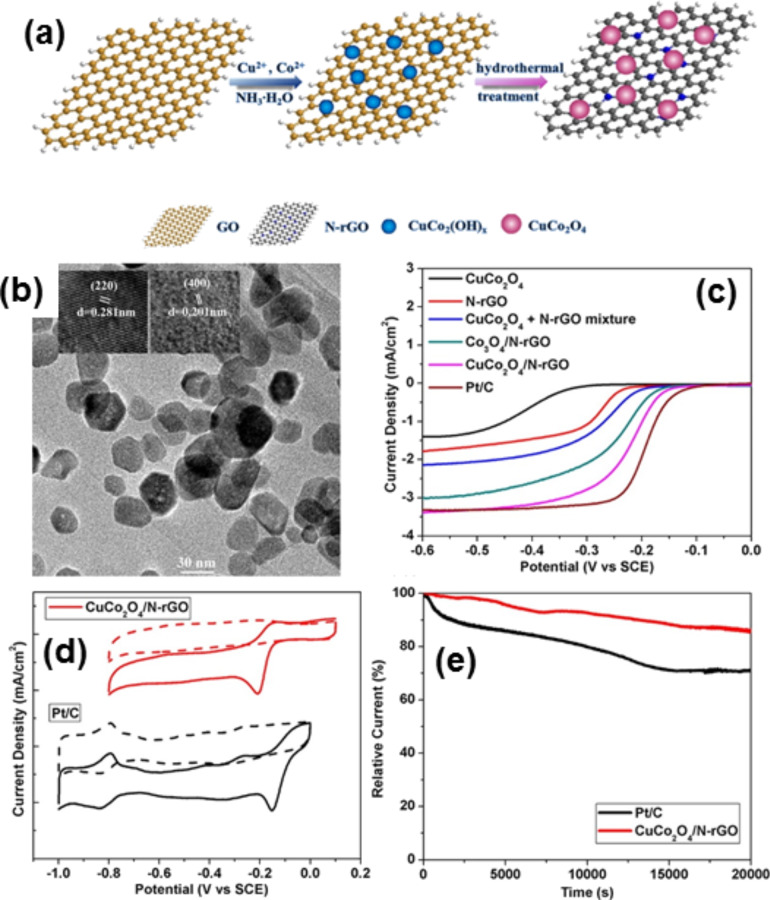
(a) Schematic diagram to illustrate the two-step solvothermal preparation of the CuCo_2_O_4_/N-rGO hybrid catalyst. (b) High-magnification TEM images of the resulting CuCo_2_O_4_/N-rGO hybrid (insets show HRTEM images of one CuCo_2_O_4_ nanoparticle). (c) Linear sweeping voltammograms (LSVs) of the CuCo_2_O_4_/N-rGO hybrid, CuCo_2_O_4_ + N-rGO mixture, Co_3_O_4_/N-rGO hybrid, CuCo_2_O_4_, N-rGO, and Pt/C in an O_2_-saturated 1 M KOH solution at 1,600 rpm. (d) CVs of CuCo_2_O_4_/N-rGO and Pt/C in an O_2_-saturated (solid line) or N_2_-saturated (dashed line) 1 M KOH solution. (e) Current−time (*i*–*t*) chronoamperometric responses for ORR on CuCo_2_O_4_/N-rGO and Pt/C catalysts at −0.3 V in an O_2_-saturated 1 M KOH solution at a rotational rate of 200 rpm. Reprinted (adapted) with permission from [309], copyright 2013 American Chemical Society.

Lee et al. shows that NiCo_2_O_4_ outperformed Co_3_O_4_–graphene hybrids in terms of the onset potential and current densities. This property is attributed to the fact that the incorporation of Ni cations into the octahedral sites of the spinel crystal structure enhances the electrical conductivity as well as to the creation of new active sites with much lower activation energy [303]. 3D graphene not only increased the conductivity of NiCo_2_O_4_, but can also offer effective buffering to accommodate the lithiation-induced stress which is beneficial to lithium storage and cycling stability [305]. The NiCo_2_O_4_–graphene is an attractive electrode material for supercapacitors [306–307]. NiCo_2_S_4_–graphene hybrids synthesised by a one-pot solvothermal strategy are used for ORR and ORE [308]. Ning et al. prepared, for the first time, spinal CuCo_2_O_4_ NPs on N-doped rGO. This was revealed to be an efficient electrocatalyst, produced by a two-step solvothermal method which exhibits ORR catalytic activity [309] (Figure 10). For the synthesis of the CuCo_2_O_4_/N–rGO hybrid, GO was reacted with Co(OAc)_2_ and Cu(OAc)_2_ in a 2:1 molar ratio at 80 °C in ethanol/water/ammonia, which led to the selective formation of the hybrid (Figure 10a). In this work, the detailed study shows that the CuCo_2_O_4_/N–rGO hybrid exhibits higher ORR catalytic activity than the separated counterparts like CuCo_2_O_4_ or N-rGO, mixtures of CuCo_2_O_4_ and N-rGO or Co_3_O_4_/N-rGO. The linear sweeping voltammograms (LSVs) of CuCo_2_O_4_/N-rGO, Co_3_O_4_/N-rGO, CuCo_2_O_4_ + N-rGO, CuCo_2_O_4_, and N-rGO suggest that CuCo_2_O_4_/N-rGO outperforms the other catalysts in terms of disk current density and half-wave potential in an O_2_-saturated 1 M KOH solution at a rotational rate of 1,600 rpm (Figure 10c). The hybrid gives an ORR peak at −0.20 V in the O_2_-saturated solution at the Pt/C electrode. The stability of the CuCo_2_O_4_/N-rGO hybrid was tested and it was observed that the hybrid affords superior durability to the commercial Pt/C catalyst.

Over the past years another spinal-type ferrite having the general formula MFe_2_O_4_ (M = Mn, Fe, Co, Ni, Cu) has attracted increased attention because of its large application as a catalyst. Spinal ferrites have interesting magnetic, magneto-resistive and magneto-optical properties. The high density magnetic storage is derived from the large surface area-to-volume ratio on the nanometre scale, and these materials have super-paramagnetic behaviour. These spinal ferrites are considered to be promising anode materials for LIBs given their high specific capacity which is typically 2–3 times higher than that of the graphite anode used in the commercial LIBs. Bai et al. have used a one-pot solvothermal method for the synthesis of MFe_2_O_4_ (M = Mn, Zn, Co, Ni) through the simultaneous reduction of GO and growth of MFe_2_O_4_ [310]. In this process, the size of the MFe_2_O_4_ microsphere can be controlled by adjusting the metal ion concentration. This hybrid displays a large adsorption capacity and photocatalytic activity towards RhB and MB and can be easily separated by a magnet. Among all the ferrites of the form MFe_2_O_4_ (M = Mn, Fe, Co, Ni, Cu), manganese ferrite (MnFe_2_O_4_) has been found to exhibit usually large capacitance. A MnFe_2_O_4_–rGO hybrid which shows higher catalytic performance than pure MnFe_2_O_4_ to activate peroxymonosulfate (PMS) to various oxidatively degraded organic pollutants in water, has been prepared by a facile approach [311].

Cai et al. have synthesised MnFe_2_O_4_–graphene hybrids by immobilising the MnFe_2_O_4_ microspheres on the graphene nanosheets by a solvothermal route for supercapacitance application [312]. This hybrid is also used for the purification of contaminated water by removal of glyphosate [313] and efficient removal of arsenic from water [314]. GO–MnFe_2_O_4_ nanohybrids are used for removal of lead and arsenic from water [314].

Another material in the family of spinal-type ferrites is magnetic cobalt ferrite (CoFe_2_O_4_) which was proposed for biomedical applications. The hybrid material shows good magnetism and could be separated from the solution by using a magnet. This hybrid has been prepared by a solvothermal method, a facial vapour diffusion method [315], and by a hydrothermal method [316]. CoFe_2_O_4_–graphene hybrids show improved electrochemical properties and are used as binder-free anode materials for LIBs [317–319]. Yao et al. have prepared CoFe_2_O_4_–graphene hybrids by chemical precipitation of Fe and Co precursors and reduction of GO in a hydrazine hydrate solution; this hybrid has been used for the degradation of phenol [320].

**Figure 11:**
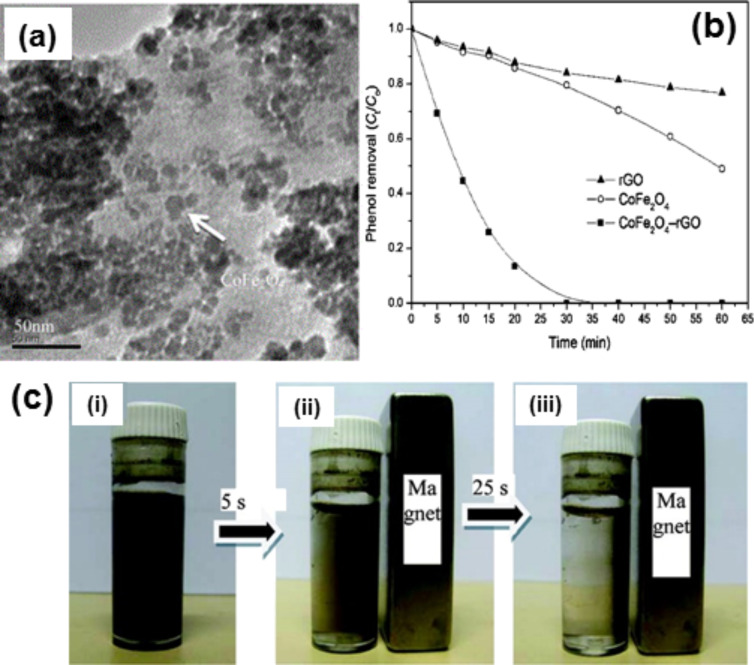
(a) TEM image of CoFe_2_O_4_−rGO hybrid. (b) Phenol degradation using CoFe_2_O_4_−rGO/PMS (reaction conditions: [phenol] = 20 mg/L, [PMS] = 0.3 g/150 mL, [catalyst] = 10 mg/150 mL). (c) Photographs of the separation and redispersion processes of CoFe_2_O_4_−rGO: (i) without external magnetic field, and (ii, iii) with external magnetic field. Reprinted (adapted) with permission from [320], copyright 2012 American Chemical Society.

Li et al. prepared graphene-functionalised CoFe_2_O_4_ by a facile hydrothermal method using inorganic salts with the thermal exfoliation of GSs. This hybrid is an effective absorbent for removing methyl orange from water [321]. Yao et al. prepared CoFe_2_O_4_–graphene hybrid materials by in situ chemical deposition and a reduction process which have catalytic performance in the heterogeneous activation of peroxymonosulfate (PMS) of phenol [320] (Figure 11).

A nitrogen absorption isotherm was used to estimate the pore size of the hybrid to be about 1.7–5.9 nm. The catalytic study of the Co_2_Fe_2_O_4_–graphene hybrid shows that the hybrid shows better catalytic activity than pure CoFe_2_O_4_ (Figure 11b). The Co_2_Fe_2_O_4_–graphene hybrids can be easily removed from the reaction solution by applying an external magnetic field (Figure 11c). CoFe_2_O_4_–graphene hybrids are used for photodegradation of MB [322–323], degradation of plasticizers [324] and the decomposition of phenol [320,325]. This material is also used as an absorbing material [315]. The bicomponent CoO/CoFe_2_O_4_ hybridised with N-doped graphene provides a simple and efficient way to configure hybridised electrode materials with high lithium storage capacity [326]. The authors used a one-step hydrothermal method followed by annealing for the successful fabrication of the bicomponent CoO/CoFe_2_O_4_. Furthermore, this hybrid has been used for microwave absorption applications and as electromagnetic wave absorbers [327]. Li et al. developed a facile one-pot polyol strategy to fabricate sandwich structures of graphene nanosheets decorated with CoFe_2_O_4_ superparamagnetic NCs [327]. The enhancement of the electromagnetic wave absorption properties of CoFe_2_O_4_ by the introduction of graphene is due to the synergistic effect between the remarkable magnetic loss from the superparamagnetic CoFe_2_O_4_ nanocrystals and high electric loss from the lightweight graphene. For LIB applications, graphene-hybridised CoFe_2_O_4_ [318] and Co_3_Sn_2_@Co [328] have been widely used.

ZnFe_2_O_4_ is an important, promising anode candidate for LIB applications due to its low discharge potential and high theoretical specific capacity of 1072 mAh·g^−1^ [329]. The ZnFe_2_O_4_-graphene hybrid films are directly usable anodes for rechargeable lithium half-cells without adding any polymer or conductive additives [330]. ZnFe_2_O_4_ is a magnetic semiconductor material, thus ZnFe_2_O_4_-based catalysts can be magnetically separated in the suspension system. ZnFe_2_O_4_–graphene hybrids prepared by the hydrothermal method show higher catalytic performance [331–332]. The improvement of the catalytic performance of the hybrid is due to the fast photogenerated charge separation and transfer due to the high electron mobility of GSs, larger light absorption, and high specific surface area of the hybrid [332]. Song et al. have prepared a ZnFe_2_O_4_–graphene hybrid by a self-assembly method for application in LIBs [333]. The hybrid acts as a catalyst for decolourisation of various dyes, elimination of the organic pollutant under visible light irradiation, and the magnetic properties of the sample helps for the easy separation from the solution [334].

**Figure 12:**
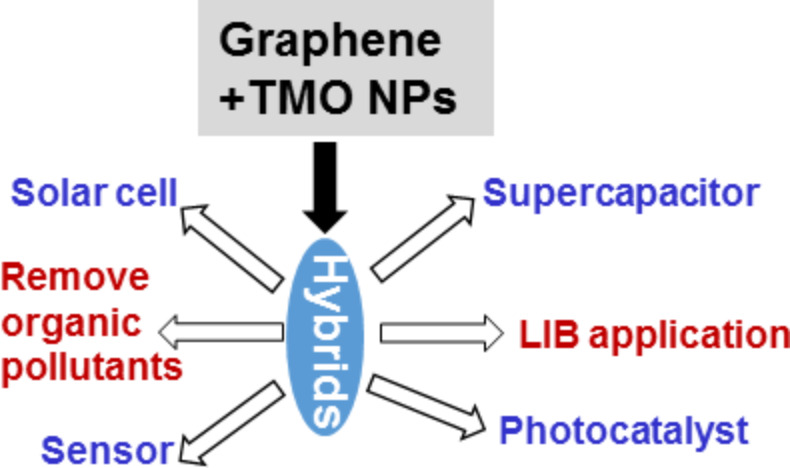
Different potential applications of graphene–TMO hybrid materials.

In summary, graphene–TMO NPs hybrids have received particular attention because of their unique properties. Due to the advantageous properties of these graphene–TMO hybrids, they have wide-spread application potential as photocatalysts, anode materials in LIBs, solar cells, sensors, diodes and also for the removal of organic pollutants from water (Figure 12).

A multitude of synthesis methods have been introduced by different research groups for the synthesis of the hybrids, among those, hydrothermal methods, sol–gel processes, chemical synthesis, and microwave-assisted growth have drawn the most attention in top-down approaches, while in bottom-up approaches, CVD and arc discharge methods have been mostly used. Depending on the application need, hybrids with different structures have been prepared by selecting the appropriate synthesis process (Table 1).

**Table 1:** Important hybrid material synthesis procedures and their potential applications.

Hybrid material	Synthesis procedure	Applications	Structure	Ref.

TiO_2_–graphene	solvothermal process	photocatalytic activity	NPs on GS	[335]
	self-assembly	photocatalytic and electrochemical activity	3D hydrogel	[89]
		LIBs	NPs	[92,98]
	hydrothermal process	photocatalytic activity	graphene-wrapped NPs	[72]
		DSSCs	NPs on GS	[87]
	chemical synthesis	LIBs	paper	[96]
		self-cleaning application	graphene-loaded thin film	[102]
	calcination process	photocatalytic activity	graphene-encapsulated hollow nanospheres	[97]
	microwave-assisted technique	supercapacitors	NPs	[86]
	reduction-hydrolysis technique	photocatalytic activity	sandwich	[90]
	molecular grafting process	DSSCs	graphene incorporated in NP films	[95]
	electrostatic deposition	photoconversion properties	multilayer films	[93]
	microwave-assisted solvothermal process	fuel cells	NPs	[101]
VO_2_–graphene	chemical synthesis	LIBs	ribbons	[111]
	layer-by-layer process	enhanced optical response	films	[109]
	CVD/Magnetron sputtering	flexible thermochomic window	films	[117]
	chemical synthesis	LIBs	graphene-coated NPs	[110,112]
	hydrothermal process	electrochemical capacitor	NPs	[108]
		LIBs	nanotube/graphene	[113]
V_2_O_5_–graphene	sol–gel process	LIBs	incorporation of GS in nanoribbons	[114]
	solvothermal process	LIBs	porous NPs	[116]
	self-assembly process	LIBs	hollow microspheres, nanorods	[107]
	solution-phase synthesis	LIBs	NPs	[115]
Cr_2_O_3_–graphene	pyrolysis of chromium/urea coordinated compound	catalyst (ORR)	rGO-supported NPs	[118]
	chemical synthesis	capacitance	NP-decorated rGO	[119]
MnO_2_–graphene	self-assembly	supercapacitors	graphene-wrapped honeycomb NPs	[141]
	layer-by-layer assembly	LIBs	thin films	[27]
	modified Hummers method and glucose reduction	oxidative decomposition of MB	NPs	[127]
	vacuum filtration process	flexible supercapacitor	quasi-2D ultrathin nanosheet	[140]
	chemical synthesis	supercapacitor	foams	[139]
Mn_3_O_4_–graphene	hydrothermal process	supercapacitor	nanorods on GS	[138]
		supercapacitor	NP anchored rGO	[126]
		carbon dioxide adsorption	porous material	[129]
		LIBs	NPs	[124]
	hydrothermal self-assembly method	supercapacitor	3D network	[131]
	chemical synthesis	catalyst (ORR)	NPs	[135]
		LIBs	NPs on rGO	[120,136–137]
		catalyst (decomposition of organic pollutants)	NPs	[128]
	gel-like film synthesis	LIBs	film	[122]
	ion exchange followed by calcination	capacitance	NPs distributed on the surface of rGO	[133]
	two-step liquid phase procedure	LIBs	NPs integrated with graphene	[130]
	deposition/precipitation method	elemental mercury capture	NPs	[134]
	ultrasound-assisted synthesis	LIBs	nanosheets	[123]
	gel formation and electrochemical reduction	electrochemical properties	paper	[132]
	chemical synthesis	electrocatalysts for vanadium redox flow batteries	coupling between the components	[125]
MnO–graphene	hydrothermal process	LIBs	nanosheets	[121]
Fe_3_O_4_–graphene	combined hydrothermal self-assembly, freeze-drying and thermal treatment	electrocatalyst (ORR)	3D aerogel	[147]
	supercritical drying and carbonizing hydrogel precursors	enzyme immobilisation	aerogels	[148]
	kirkendall process	LIBs	core–shell nanohollow	[161]
	filtration process	electrochemical actuators	paper	[158]
	vacuum filtration and thermal reduction process	LIBs	flexible films	[146]
	solvothermal treatment	LIBs	graphene-coated NPs	[162]
	solution chemistry	regenerative adsorbent	NPs decorated on rGO	[167]
	chemical synthesis	LIBs	NP-anchored graphene nanosheets	[163]
Fe_2_O_3_–graphene	hydrothermal process	LIBs and arsenic removal	network	[154]
		nonenzymatic H_2_O_2_ biosensors	NPs decorated on rGO	[155]
	solvothermal induced self-assembly	LIBs	aerogels	[166]
Co_3_O_4_–graphene	chemical synthesis	catalyst (ORR and ORE)	NPs on graphene	[170]
		LIBs	Nanowall arrays on rGO	[169]
		LIBs	Graphene-anchored NPs	[178]
		catalyst (ORR)	nanosheet	[176]
	hydrothermal process	oxidation of olefins and alcohols	sandwich	[174]
CoO–graphene	chemical synthesis	ORR	NPs assembled on graphene	[189]
		LIBs	nanosheets	[187]
	assembly by electrostatic forces	LIBs	graphene-encapsulated NPs	[185]
NiO–graphene	electrophoretic deposition and chemical bath deposition	electrochromic performance	films	[196]
	chemical process	NO_2_ sensors	2D nanosheets	[204]
	chemical bath deposition technique	supercapacitors	3D foams	[203]
	microwave-assisted synthesis	supercapacitors	graphene-wrapped NPs	[206]
CuO–graphene	spin-coating, Magnetron sputtering	blocking layer and O_2_ ion storage	multilayer	[217]
	vacuum filtration and hydrothermal reduction	LIBs	lamellar paper	[336]
	hydrothermal method	electrochemical capacitors	leaf-like NPs on GS	[223]
Cu_2_O–graphene	hydrothermal process	NO_2_ sensor	mesocrystals	[214]
	chemical reduction method	electrochemical sensor (glucose and H_2_O_2_)	graphene-wrapped NPs	[211]
ZnO–graphene	chemical synthesis	white LEDs	quasi-QDs	[224]
	hydrothermal method	photocatalytic activity	nanomesh	[233]
	hydrothermal process with surface modification	wave absorption	graphene-wrapped hollow NPs	[228]
	functionalisation of NPs followed by hydrothermal method	photodetector	core–shell	[226]
	atomic layer deposition, CVD	sensor (formaldehyde)	films	[266]
	thermal evaporation technique	UV photodetector	NWs on 3D graphene foam	[275]
	freeze-drying, subsequent heat treatment method	LIBs	NPs anchored on graphene	[240]
NiCo_2_O_4_–graphene	freeze-drying and hydrothermal reduction	supercapacitors	3D mesoporous	[307]
	polyol and thermal annealing	electrocatalyst (ORR)	nanosheets	[299]
	hydrothermal method followed by calcination	supercapacitors	nanorods and nanobundles	[306]
MnCo_2_O_4_–graphene	chemical synthesis	catalyst (ORR)	NPs on GS	[29]
CoFe_2_O_4_–graphene	chemical synthesis	LIBs	films	[318]
	solvothermal route	LIBs	sandwich	[317]
		catalyst (ORR)	NPs on GS	[309]
ZnFe_2_O_4_–graphene	hydrothermal synthesis	LIBs	octahedrons	[329]
	deposition/precipitation	photocatalyst	multiporous microbricks	[332]
LiFePO_4_–graphene	catalyst-assisted self-assembly method	LIBs	graphene-embedded NPs	[288]
	chemicals synthesis	LIBs	sandwich	[287]

## Conclusion

Since the first experimental demonstration of single sheet graphene in 2004, the field of graphene-related research has grown at an outstanding speed. Graphene has a unique morphology, large surface area, and extraordinary electronic properties. Graphene has been synthesised by various methods and its chemical structure can be modified by changing the fabrication methods and also by combining with various NPs. Such materials are produced to meet the increasing demands of high-performance materials in electronic devices.

Enhanced knowledge on the surface chemistry and functionalisation of the graphene surface, and also on the improvement in the colloidal synthesis of NPs, will lead to a wide range of potential applications related to the preparation of graphene–NPs hybrid materials by the incorporation of various NPs. The synthesis of graphene-based composites also requires a large amount of graphene, preferably with surface modification. By tuning the surface chemistry of graphene, the fluorescent properties can be changed accordingly which enables its potential application in photovoltaics, sensors and also in bio-imaging. The number of publications related to graphene and graphene-based materials has increased exponentially in recent years. However, there is still need for the scientific community to develop graphene-based models, hybrid materials and devices, and their possible applications in various fields. By improving the quality of the synthesised graphene, better quality hybrids can be prepared for different applications.

## Perspectives

Knowledge of GO chemistry will provide valuable input about its reactive properties and the properties of the graphene derived from it. Controlled methods of graphene synthesis are particularly important for the controlled growth of layers, which still remains to be established. Regarding the fabrication of graphene NP hybrids, the key obstacle lies in the reproducibility of the structure. For the synthesis of graphene-encapsulated NPs, the difficulty is that the thin graphene layer is not ideally formed as a uniform layer on all the NPs. Thus, the design and synthesis of new graphene-based nanostructures and architectures with uniform structure will be an important task for the future. It is important to mention that no such unique analysis technique has been introduced which can confirm that all the NPs are uniformly coated. In most reports directed at graphene-encapsulated structures, a quasi-core–shell structure was obtained instead of the desired core–shell structure.

By achieving a high level of uniformity and reproducibility of graphene–NP hybrids, the electrical properties of the devices can be optimised which will lead to higher sensitivity and selectivity. Inexpensive and large-scale fabrication of defect-free graphene is still a challenge due to the existence of defects or oxidation sites in graphene, which is partially restored in graphene derived from GO. However, there is no doubt that more efficient synthetic strategies for graphene and graphene-based hybrids will be developed in an economically feasible quantity, leading to the commercialisation of graphene in the near future. Some research shows that the surfactants and the reductants used for the synthesis of the hybrid materials have a detrimental effect. In this sense, the biocompatibility and toxicity of the chemicals should be investigated to enable application of the hybrid materials in biomedical applications. Most of the applications of TMO–graphene hybrid materials are in LIBs, although further study is required to gain control over the phase and morphology of the NPs for this application. A prerequisite is a well-mastered growth synthesis to obtain a well-defined uniform structure of graphene. Control over the interface and interaction between NPs and graphene is necessary to enhance the performance of LIBs. Much attention is devoted to the application of graphene–NP hybrids in supercapacitor applications but more research is necessary. During the charge–discharge process, the graphene layers become aggregated, which degrades the electrode material. In some cases, the electrode materials are contaminated by impurities introduced from the incomplete reduction of GO. When using graphene–TMO hybrids as a photocatalyst, the improved performance in the catalytic properties is generally ascribed to the extended absorption and faster charge transfer in graphene as compared to TMOs. Therefore, research on the interfacial status of the hybrid is important to obtain a detailed understanding of the origin of the improvement.

Further challenges exist in the application of graphene–TMO NP hybrids on the industrial scale. Some advanced applications of the graphene-based hybrids require particular understanding of the contact between the surface of the graphene and the NPs, which will have a direct impact on the properties of the hybrid. Despite the enormous efforts and huge attention, a significant breakthrough application of graphene and graphene-based materials in energy harvesting systems has not been achieved to date, but solutions to the key challenges appear within reach. In view of the manifold of potential applications, each having different requirements regarding material properties, it can be anticipated that the research on graphene–TMO NP hybrid materials will have a very bright future.
